# Disentangling nigral and putaminal contribution to motor impairment and levodopa response in Parkinson’s disease

**DOI:** 10.1038/s41531-022-00401-z

**Published:** 2022-10-14

**Authors:** Nils Schröter, Michel Rijntjes, Horst Urbach, Cornelius Weiller, Martin Treppner, Elias Kellner, Wolfgang H. Jost, Bastian E. A. Sajonz, Marco Reisert, Jonas A. Hosp, Alexander Rau

**Affiliations:** 1 Department of Neurology and Clinical Neuroscience, Medical Center–University of Freiburg, Faculty of Medicine, University of Freiburg, Freiburg, Germany; 2 Department of Neuroradiology, Medical Center–University of Freiburg, Faculty of Medicine, University of Freiburg, Freiburg, Germany; 3 Institute of Medical Biometry and Statistics, Medical Center - University of Freiburg, Faculty of Medicine, University of Freiburg, Freiburg, Germany; 4 Department of Medical Physics, Medical Center – University of Freiburg, Faculty of Medicine, University of Freiburg, Freiburg, Germany; 5 Parkinson-Klinik Ortenau, Wolfach, Germany; 6 Department of Stereotactic and Functional Neurosurgery, Medical Center - University of Freiburg, Faculty of Medicine, University of Freiburg, Freiburg, Germany; 7 Department of Diagnostic and Interventional Radiology, Medical Center - University of Freiburg, Faculty of Medicine, University of Freiburg, Freiburg, Germany

**Keywords:** Parkinson's disease, Parkinson's disease, Parkinson's disease

## Abstract

The extent to which the degeneration of the substantia nigra (SN) and putamen each contribute to motor impairment in Parkinson’s disease (PD) is unclear, as they are usually investigated using different imaging modalities. To examine the pathophysiological significance of the SN and putamen in both motor impairment and the levodopa response in PD using diffusion microstructure imaging (DMI). In this monocentric retrospective cross-sectional study, DMI parameters from 108 patients with PD and 35 healthy controls (HC) were analyzed using a voxel- and region-based approach. Linear models were applied to investigate the association between individual DMI parameters and Movement Disorder Society Unified Parkinson’s Disease Rating Scale-Part 3 performance in ON- and OFF-states, as well as the levodopa response, controlling for age and sex. Voxel- and region-based group comparisons of DMI parameters between PD and HC revealed significant differences in the SN and putamen. In PD, a poorer MDS-UPDRS-III performance in the ON-state was associated with increased free fluid in the SN (b-weight = 65.79, *p* = 0.004) and putamen (b-weight = 86.00, *p* = 0.006), and contrariwise with the demise of cells in both structures. The levodopa response was inversely associated with free fluid both in the SN (b-weight = −83.61, *p* = 0.009) and putamen (b-weight = −176.56, *p* < 0.001). Interestingly, when the two structures were assessed together, the integrity of the putamen, but not the SN, served as a predictor for the levodopa response (b-weight = −158.03, *p* < 0.001). Structural alterations in the SN and putamen can be measured by diffusion microstructure imaging in PD. They are associated with poorer motor performance in the ON-state, as well as a reduced response to levodopa. While both nigral and putaminal integrity are required for good performance in the ON-state, it is putaminal integrity alone that determines the levodopa response. Therefore, the structural integrity of the putamen is crucial for the improvement of motor symptoms to dopaminergic medication, and might therefore serve as a promising biomarker for motor staging.

## Introduction

The degeneration of dopaminergic neurons in the substantia nigra (SN) is the hallmark of Parkinson’s disease (PD) and is of pathophysiological importance to the clinical course of this condition^1^. By the time clinical motor symptoms have manifested, at least 30% of SN dopaminergic cells and up to 50% of their axon terminals that project to the putamen have perished^2,3^. This discrepancy between the pronounced loss of dopaminergic terminals versus only mild clinical deficits is partially explained by various compensatory mechanisms, such as those afforded by serotonergic neurons^4^. The response to levodopa varies widely between individuals in PD, and is highly relevant not only in terms of individual patient therapy but also as a diagnostic marker for clinicians^5,6^. However, the cause of a declining response to levodopa is currently controversial, and factors such as the loss of dopaminergic neurons in the SN and/or their terminals in the putamen, or diffuse Lewy body pathology, have been discussed as possible underlying mechanisms^4,7,8^.

The degeneration of neuromelanin-containing neurons in the SN pars compacta versus the associated loss of putaminal terminals cannot be delineated using conventional macrostructural imaging techniques^9^. Thus, dopamine-transporter single-photon emission computed tomography (SPECT) has been established as the gold standard for in vivo assessment of nigrostriatal pathology^10^. However, because this technique only determines the functional integrity of striatal dopaminergic synapses, it merely serves as indirect evidence of SN pathology^10^. On the other hand, diffusion-based MRI techniques are a promising means of assessing the structural integrity of the SN and putamen in vivo. The microstructural composition can be approximated by mesoscopically distinguishing between different anatomical compartments on the basis of their diffusion properties. Compared to classical diffusion tensor imaging (DTI) indices, biophysical-based models such as neurite orientation dispersion and density imaging (NODDI) and diffusion microstructure imaging (DMI) offer more specific measures of tissue integrity, with better interpretability^11,12^. Similar to the NODDI approach of detecting gray matter changes in PD, DMI detects degenerating neuronal somata and terminals of unmyelinated axons by way of a decrease in the parameter “V-extra”, while degenerating dendrites and myelinated axons are detected by a decrease in “V-intra”. Moreover, an increase in free interstitial fluid resulting from the loss of axons and cell bodies leads to an elevated “V-CSF”^13^. In contrast to NODDI, DMI is not constrained to hard a priori assumptions and therefore more suited to the assessment of pathologically altered microstructure^12^, where it has already been successfully applied in clinical research^14–16^.

This study therefore aimed to examine microstructural alterations to the SN and putamen in patients with PD and assess their pathophysiological relevance to motor impairment and levodopa response. We hypothesized that: (1) PD-related neurodegeneration leads to the demise of somata and their dendrites in the SN pars compacta, as well as an increase in free interstitial fluid in the SN and putamen, and this would be reflected by corresponding microstructural alterations in DMI findings, and that (2) these microstructural alterations are associated with motor impairment and levodopa responsiveness, and (3) the SN and putamen each contribute independently to motor impairment and the degree of response to levodopa.

## Results

### Voxel-wise group comparison of DMI parameters

**Fig. 1:**
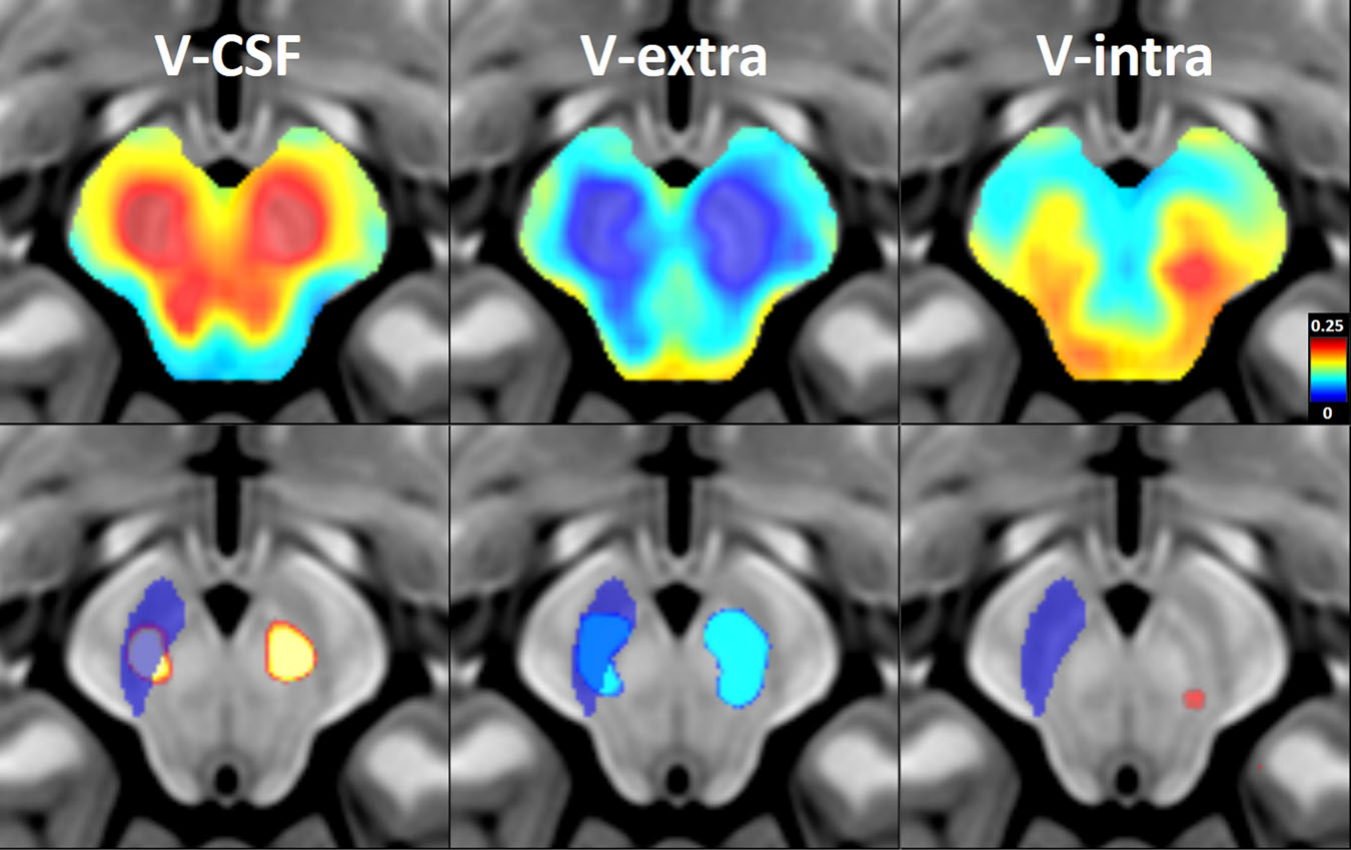
Voxel-wise group comparison of nigral microstructure between patients with PD and healthy controls. Voxel-wise group comparison of DMI parameters between patients with PD (*n* = 108) and healthy controls (*n* = 35) superimposed on a T1w MRI template in standard space. The analysis controlled for age and sex, and a 5% false discovery rate (FDR) was applied. *Top row:* Standardized regression coefficients were extracted from regression models in PD and HC groups (with the covariates “age” and “sex”). Color-coding indicates coefficient values as a measure of effect size of the factor “Parkinson’s disease” on DMI-parameters (hot colors: positive effect size vs. cold colors: negative effect size). *Bottom row:* Significant results of the voxel-wise group comparisons with a 5% FDR. The right SN is shown in blue. The left side of the image corresponds to right side of the patient’s body. *DMI* diffusion microstructure imaging, *SN* substantia nigra, *FDR* false discovery rate, *V-CSF* compartment of free interstitial fluid, *V-extra* compartment of somata and unmyelinated axons, *V-intra* intra-axonal and dendritic compartment.

**Fig. 2:**
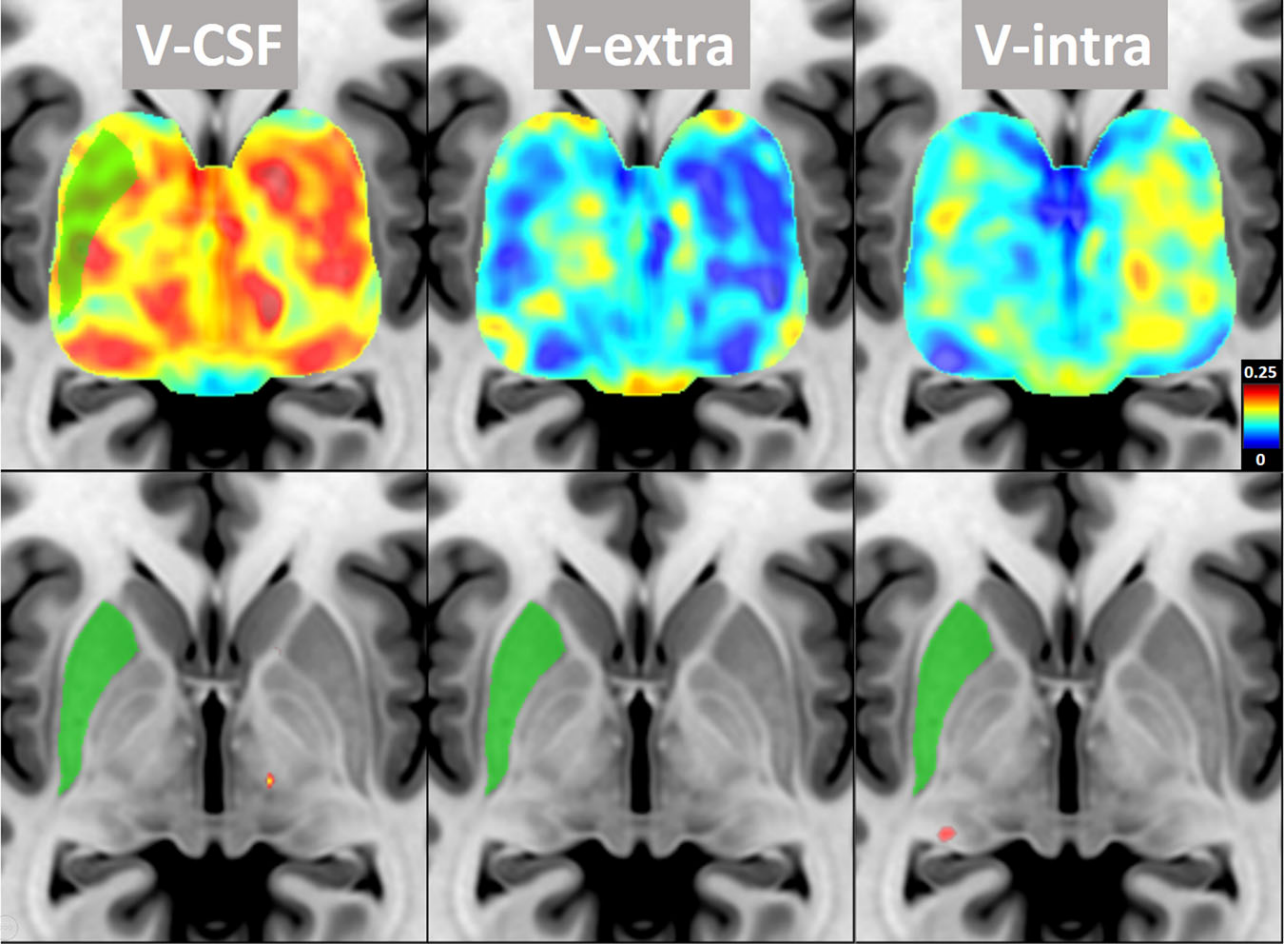
Voxel-wise group comparison of putaminal microstructure between patients with PD and healthy controls. Voxel-wise group comparison of DMI parameters between patients with PD (*n* = 108) and healthy controls (*n* = 35) superimposed on a T1w MRI template in standard space. The analysis controlled for age and sex, and a 5% false discovery rate (FDR) was applied. *Top row:* The standardized regression coefficients were extracted from regression models both in PD and HC groups (with the covariates “age” and “sex”). Color-coding indicates the coefficient values as a measure of effect size of the factor “Parkinson’s disease” on DMI-parameters (hot colors: positive effect size vs. cold colors: negative effect size). *Bottom row:* Significant results of the voxel-wise group comparisons with a 5% FDR. The right putamen is shown in green. The left side of the image corresponds to right side of the patient’s body. *DMI* diffusion microstructure imaging, *FDR* false discovery rate, *V-CSF* compartment of free interstitial fluid, *V-extra* compartment of somata and unmyelinated axons, *V-intra* intra-axonal and dendritic compartment.

Group differences in cerebral microstructure between PD and HC were first assessed using a voxel-based analysis. Extensive alterations in the free fluid fraction and the cellular and extracellular compartment (V-CSF and V-extra) were observed in the midbrain and basal ganglia, while mixed effects were observed in the intra-axonal and dendritic compartment (V-intra) (see Figs. 1 and 2).

To examine the direction (increase versus decrease) and spatial distribution of these alterations, we extracted the standardized regression coefficients (beta) of the factor V-CSF and V-extra as a measure of effect size. A widespread increase in V-CSF with a corresponding decrease in V-extra was observed in the midbrain and putamina (Figs. 1 and 2).

After FDR correction, significant group differences were found in the tegmental midbrain, with higher V-CSF and lower V-extra readings in PD compared to HC (Fig. 1). This area mainly corresponded anatomically to the SN (Fig. 1).

Extensive alterations were also present in the putamen, with an increase in V-CSF, a decrease in V-extra, and minor alterations to V-intra. However, no significant differences were observed after FDR correction (Fig. 2).

### ROI-based group comparison of DMI parameters in the substantia nigra and putamen

**Fig. 3:**
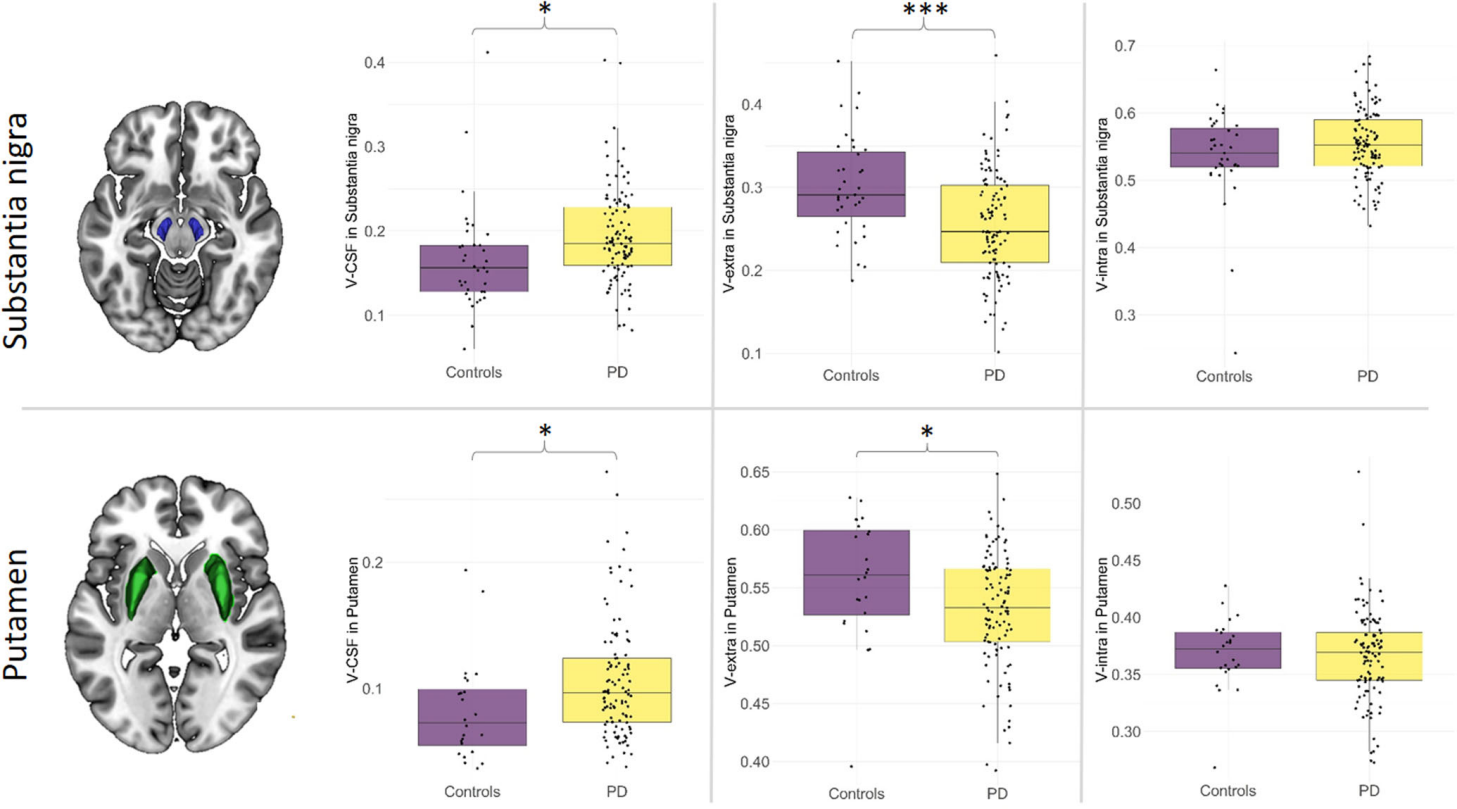
ROI-based group comparison of nigral and putaminal microstructure between patients with PD and healthy controls. Group comparisons of microstructural compartments (V-extra, V-CSF, V-intra) in the SN (*top row*) and putamen (*bottom row*) between PD and HC groups. Dots represent individual subject values, while boxplots show group median, quartiles, minimum and maximum values. Significance threshold: *p < 0.05, ***p < 0.001. PD Parkinson’s disease.

In the second hypothesis-based step, DMI parameters in the SN and putamen were determined by ROIs defined via a probabilistic atlas^17,18^ and then compared between PD and HC groups. In line with the exploratory voxel-wise results, ROI-based comparison of nigral microstructure revealed significant increases in the free fluid fraction (V-CSF, *p* = 0.013; df: 139; t = 1.887; Cohen’s d: −0.477) and corresponding decreases in cell bodies (V-extra, *p* < 0.001; df: 139; t = −3.228; Cohen’s d: 0.717), while the intra-axonal and dendritic compartments remained unchanged (V-intra, *p* = 0.080; df: 139; t = 1.528; Cohen’s d: −0.327). Similarly, there was an increase in the free fluid fraction in the putamen (V-CSF, *p* = 0.010; df: 128; t = 1.887; Cohen’s d: −0.519), while the cellular fraction was reduced (V-extra, *p* = 0.010; df: 128; t = −1.813; Cohen’s d: 0.513) the intra-axonal compartment was unchanged (V-intra, *p* = 0.834; df: 128; *t* = −0.420; Cohen’s d: 0.047) (Fig. 3).

A weakly positive Pearson’s correlation coefficient for V-CSF was observed between the SN and putamen (R = 0.36, *p* < 0.001), whereby V-CSF showed a larger range in the SN (0.06 to 0.40) compared to that of the putamen (0.04 to 0.27). Upon comparison of the ROIs ipsi- and contralateral to the more affected side, no group differences in DMI metrics were observed (Supplementary Fig. 2).

### ROI-based analysis of the association between nigral and putaminal microstructure and clinical parameters

As shown in Figs. 1 and 2, V-CSF and V-extra represent the same process with the opposite direction effect, which is reflected in a moderately negative Pearson’s correlation coefficient between V-CSF and V-extra of SN (R = −0.6, *p* = <0.001), while minimal significant effects on V-intra were observed after FDR correction. Since the three-compartment model defines “1 = V-CSF + V-intra + V-extra”, and the increase in V-CSF was inversely regulated compared to the reduction in V-extra, the association between the microstructural compartments and clinical values are only shown for V-CSF (see Supplement for V-extra and V-intra data). All linear regression models were fitted with age and sex as covariates.

**Fig. 4:**
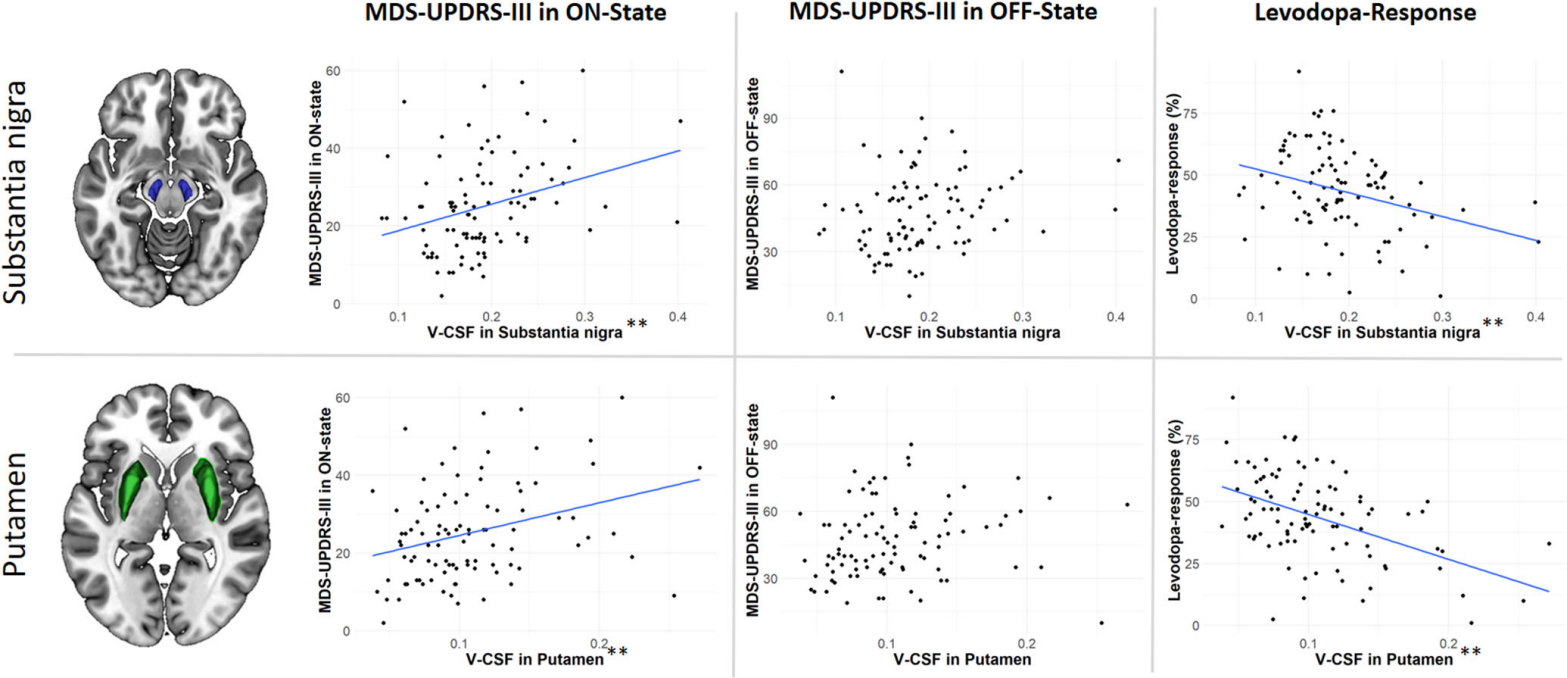
Association of nigral and putaminal microstructure with motor impairment and levodopa response. Association between V-CSF in the substantia nigra and putamen with clinical parameters. Dot plots illustrate the association between nigral V-CSF (top row) and putaminal V-CSF (bottom row) with MDS-UPDRS III in the ON-state, MDS-UPDRS-Part III in OFF-state, and the levodopa response. Each dot represents a single patient. A regression line is shown in blue. Significance threshold: **p < 0.01.

No association was observed between MDS-UPDRS III in the OFF-state and V-CSF in the SN or putamen. A positive association was observed between MDS-UPDRS III in the ON-state and V-CSF in the SN (b-weight = 65.79, 95% CI [21.41–110.17], *p* = 0.004) and putamen (b-weight = 86.00, 95% CI [25.71–146.28], *p* = 0.006). The levodopa response was negatively associated with V-CSF in the SN (b-weight = −83.61, 95% CI [−146.27–21.05], *p* = 0.009) and putamen (b-weight = −176.56, 95% CI [−256.67, −96.44], *p* < 0.001) (Fig. 4); all models are presented in detail in the Supplementary Material section.

To determine whether it is the loss of nigral or putaminal integrity that contributes more to motor impairment, linear models with both nigral and putaminal V-CSF were applied. No association was observed with MDS-UPDRS III in the OFF-state, whereas MDS-UPDRS III in the ON-state was associated with both nigral (b-weight = 52.36, 95% CI [7.11–97.60], *p* = 0.024, VIF 1.09) and putaminal V-CSF (b-weight = 66.64, 95% CI [5.38–127.91], *p* = 0.033, VIF 1.21). The levodopa response was negatively associated with putaminal (b-weight = −158.03, 95% CI [−239.79 to −76.27], *p* < 0.001, VIF 1.38) but not nigral V-CSF (b-weight = −54.49, 95% CI [−114.73 to 5.75], *p* = 0.076, VIF 1.16).

## Discussion

This study used diffusion microstructure imaging to investigate the role of microstructural integrity in the SN and putamen in PD. Comparison of nigral and putaminal degeneration in patients with PD versus healthy controls revealed an increase in the amount of free fluid (V-CSF) and a reduction in the cellular and extracellular compartment fractions (V-extra). These microstructural alterations were associated with poorer MDS-UPDRS III scores in the ON-state, as well as a reduced response to levodopa, whereas the latter was primarily determined by putaminal integrity.

Diffusion MRI techniques have previously been employed to investigate the SN, whereby a NODDI study in 44 patients with PD reported an elevated extracellular fluid fraction, but no effect on the neurite compartment in the SN^19^. We now build on these findings by demonstrating a reduction in the somata-associated fraction, using both a voxel-wise and a ROI-based approach. These additional findings are primarily explained by our larger sample size, and the considerably longer disease duration and more severe motor impairment in the patient cohort. Of note, while the SN can be anatomically as well as functionally divided into pars reticularis and pars compacta, we analyzed the SN as a whole to develop a biomarker more resistant to partial volume effects and atrophy due to the larger ROI^20,21^.

We observed homologous microstructural alterations in the putamen, where alterations in V-CSF and V-extra corresponded to an increase in free fluid and a reduction in the cellular and extracellular fractions. These findings are corroborated by previous studies that described a decrease in the putaminal cellular fraction^19,22^. This is likely attributable to the demise of dopaminergic terminals of unmyelinated axons, dendritic remodeling of medium spiny neurons, and impaired neuronal outgrowth that occurs early on during the course of PD^23–25^. The lack of significant group differences observed in the putamen in voxel-wise comparisons is likely due to the fact that the degenerating dopaminergic terminals and neuronal somata in the putamen account for a much smaller volume fraction than those in the SN. Accordingly, the free fluid fraction range in the SN was notably broader than that observed in the putamen. Though, as expected in view of the standardized regression coefficients, significant differences were found in the ROI-based comparison.

The close association between putaminal microstructural alterations and motor symptoms in ON-state underscores the high pathophysiological relevance of putaminal integrity. However, only minor and non-significant effects were observed in the intra-axonal and dendritic compartments in both the SN and putamen. This is primarily explained by the very small fraction these structures account for in the gray matter. These findings might seem unexpected, since neuropathological studies have shown that putaminal dopaminergic markers are lost within 4 years after diagnosis^26^. That significant effects were still observed with DMI might be explained by the fact that the putamen is considered as a holistic structure including compensatory, e.g. serotonergic mechanisms, and not only selectively the dopaminergic axis^27^.

Using free-water imaging, Ofori et al.^28^ observed a positive correlation between MDS-UPDRS III scores and free water in the posterior SN. Further to our own observations of an increased free-fluid fraction, we noted that MDS-UPDRS III scores were negatively associated with the cellular compartment. We interpret this as a correlate of cell demise that subsequently leads to motor deterioration, a notion which is supported by previous neuropathological findings that linked MDS-UPDRS III performance scores to nigral cell loss^3^.

No significant differences were observed between the sides ipsi- and contralateral to the more affected side. This is in line with a previous study assessing nigral structural integrity using neuromelanin sensitive imaging^29^. In contrast, a more significant impairment of the side contralateral to the clinically more affected side is observed in studies using striatal dopamine transporter^30,31^ or diffusion-tensor-imaging^32^. However, neuropathological data regarding this issue is sparse with only one study investigating nigral neuron count bilaterally, demonstrating a significant difference between both sides in 21 patients^33^. Thus, further studies are needed to unravel the complex basis of asymmetry in PD^34^.

Whereas strong associations between microstructure and performance in the ON-state were observed, no correlation was evident for the OFF-state. The OFF-state itself is heterogeneous in PD, since various compensatory mechanisms can take effect; this leads to a high inter-individual variance in motor symptoms, explaining why some patients already become symptomatic when nigrostriatal degeneration is comparatively slight, while others show more progressive degeneration^2,3,26^. Second, when dopaminergic medication is withheld overnight, the short levodopa response has worn off, whereas the long levodopa response with different, long lasting compensatory mechanisms still persists, leading to a decrease of 30% in MDS-UPDRS-III scores compared to dopamine-naïve OFF scores^35^. Moreover, despite withholding the medication for 24 h, a residual effect of dopamine agonists with individual heterogeneity can be assumed, which then manifests as low MDS-UPDRS-III scores in the OFF-state^36^. In contrast, the ON-state is clinically well-defined and achieved by targeting maximal dopaminergic stimulation, leading to more consistent scores. These mechanisms might therefore explain why there was no significant correlation between nigral and putaminal microstructure and the OFF-state.

The extent to which neurodegeneration in PD originates from putaminal terminals or nigral somata of dopaminergic cells is currently unknown^26,37,38^. A recent study reported that although simultaneous degeneration of nigral presynaptic terminals and a reduction in putaminal dopamine transporter integrity were observed, these alterations were not found to be associated with motor-impairment severity^8^. Our data provide evidence that the degree of structural integrity in the putamen—formed by both dopaminergic terminals and somata—influences the response to levodopa, while good motor performance in the ON-state depends on both nigral and putaminal integrity. This is primarily due to the ON-state relying on basal dopaminergic stimulation by the remaining nigral cells, as well as the effect of dopaminergic medication, which itself relies on putaminal structural integrity. The dissociation between nigral and putaminal degeneration was already shown in the MPTP (1-methyl-4-phenyl-1,2,3,6-tetrahydropyridine) macaque model where a correlation between nigral degeneration and presynaptic dopaminergic nigrostriatal PET tracers was only observed as long as more than half of the nigral cells were preserved, whereas the correlation between striatal uptake was present for all disease stages which is in line with our findings^39,40^.

While the significance of dopaminergic terminal loss has been increasingly demonstrated^41^, and there is now evidence for a strong association between putaminal microstructural alterations and the levodopa response, we additionally noted a strong impact of SN neuronal loss on general motor performance. This finding suggests that it is not only the loss of dopaminergic efferent terminals but also that of their associated somata in the SN that has a clinically relevant impact on motor symptoms in PD. Furthermore, these two factors could serve as potential targets for future treatment approaches, or as a biomarker for disease-modifying therapies.

Nonetheless, it remains controversial why patients with PD develop motor symptoms with a variable degree of advanced nigral degeneration^26^. Our data suggest that putaminal integrity has a pronounced impact on motor impairment, whereby a so-called “putaminal reserve” might be crucial for the clinical manifestation of motor symptoms. Therefore, future studies—especially neuroprotective trials such as those in patients with prodromal PD—should not only focus on the substantia nigra but also on the putamen^42^.

This study is limited by its retrospective nature. With data obtained from routine clinical practice, different raters could have potentially led to a greater variance in the clinical findings. This outcome was circumvented by only using test scores derived from formal, structured testing of the levodopa response, thus corresponding to highly standardized procedures. Also, the diagnostic accuracy associated with PD is approximately 80% in a clinical setting^43,44^. Postmortem-validated diagnoses were not available, so it cannot be excluded that patients with diseases other than PD were erroneously included. However, a large proportion of patients underwent additional diagnostic procedures, such as FDG-PET, which improves diagnostic reliability^45^. In principle, Levodopa equivalent daily dose (LED) constitutes a potential confounder in the analyses regarding dopaminergic medication and motor symptom response. However, a detailed investigation of this effect was not possible because a valid analysis was not feasible due to the retrospective setting as LED was primarily determined by the referring physicians that cover a wide range of experience in therapy of PD.

A major strength of the present study is that both the SN and putamen were investigated using the same method, namely DMI. The fact that we observed uniform effects on both these structures, in terms of both voxel-wise and ROI-based analyses, underlines the validity and reliability of the DMI approach.

Furthermore, the generalizability of our results is supported by our large representative cohort, in whom there was wide range of disease duration and motor impairment. Moreover, a non-biased approach was chosen to compare PD and HC groups by using an exclusive voxel-based analysis of the DMI parameters, and these findings were confirmed in a ROI-based approach in the second step. In contrast to previous studies that used Pearson’s correlations to assess the association between clinical parameters and multicompartment imaging, we chose linear models that corrected for age and sex, given the relevant impact of these factors on diffusion MRI values^28,46,47^.

In summary, our analysis of a large, clinically representative cohort revealed that structural alterations in the SN and putamen can be measured by DMI, and that these alterations are associated with poorer motor performance in the ON-state, as well as a reduced response to levodopa. Furthermore, both nigral and putaminal integrity were found to be required for good performance in the ON-state. Using the same methodological approach, we have also shown for the first time that the levodopa response is primarily determined by putaminal integrity. Therefore, the structural integrity of the putamen is crucial for improving the motor response to dopaminergic medication and might therefore be a promising biomarker for motor staging, as well as an intriguing target for future disease modifying therapeutics.

## Methods

### Patients

This retrospective analysis included DMI-MRI data from (I) 108 consecutive patients with PD who underwent a routine MRI between 01/2018 and 02/2021 for the evaluation of advanced Parkinson therapies or for differential diagnosis and (II) 35 healthy age- and sex-matched controls. The study was approved by the Institutional Review Board (Ethics Committee—University of Freiburg; EK 22/20) and carried out in accordance with the Declaration of Helsinki and its later amendments. Due to the retrospective nature of this study, the need for written informed consent was waived.

Patients in whom PD was either clinically established or highly probable were included in the study. Clinical diagnoses were validated by two experts in movement disorders (MR, board certified neurologist; NS > 7 years of neurology training) based on current diagnostic criteria; all available medical records were used for the consensus diagnosis^5^. Imaging data indicative of PD were available in the form of N-ω-fluoropropyl-2β-carbomethoxy-3β-(4-[^123^I]iodophenyl)nortropane ([^123^I]FP-CIT) data (41 patients), or ^18^fluorodeoxyglucose (FDG)-PET data (80 patients)^45^; 33 patients underwent both imaging procedures.

**Fig. 5:**
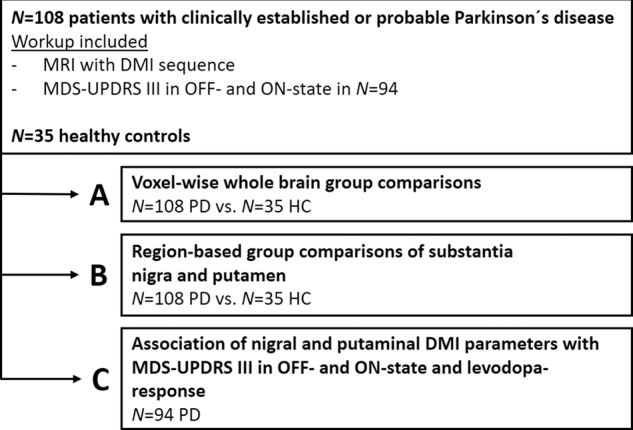
Study protocol and allocation of participants. *MDS-UPDRS III* Movement Disorder Society Unified Parkinson’s Disease Rating Scale-Part 3, *PD* Parkinson’s disease, *HC* healthy controls, *DMI* diffusion microstructure imaging.

Data derived from the Movement Disorder Society Unified Parkinson’s Disease Rating Scale-Part III (MDS-UPDRS-III) were available in 94 patients following discontinuation of dopaminergic medication for at least 24 h in the OFF-state, as well as the MDS-UPDRS-III in the ON-state one hour after supplementation of levodopa (soluble preparation; Madopar LT, Roche Pharma AG, Basel, Switzerland; 1.5 times the morning equivalent dose, with a minimum of 250 mg) dopaminergic medication^48,49^. The response to dopaminergic medication on formal testing is given in percent. Demographic and clinical data are shown in Table 1, and the study protocol is summarized in Fig. 5.

**Table 1:** Demographics and clinical characteristics of the PD patient group.

Parameter	Patients with Parkinson’s disease
*n*	108
Age (years) Mean (Range, SD)	67 (44–81, ±8)
Sex (Female/Male %)	34/66
Disease Duration (years) Mean (Range, SD)	10 (1–26, ±6)
Hoehn & Yahr Stage Mean (Range, SD)	2.9 (1–5, ±0.8)
MDS-UPDRS III, OFF state Mean (Range, SD)	48 (10–111, ±18)
MDS-UPDRS III, ON state Mean (Range, SD)	25 (2–60, ±12)
Levodopa response in % Mean (Range, SD)	44 (1–92, ±17)
Absolute levodopa response Mean (Range, SD)	22 (0–59, ±12)

*MDS-UPDRS III* Movement Disorder Society Unified Parkinson’s Disease Rating Scale-Part 3, *SD* standard deviation.

Subjects in the healthy control (HC) group had no known neurological medical condition, no neurological deficits upon clinical examination and no family history of parkinsonism. Furthermore, this group of subjects showed no neuropsychological impairment according to the Montreal Cognitive Assessment^50^. There were no group differences in terms of sex (χ^2^, *p* = 0.188) and age (t-test, *p* = 0.07) between the HC and PD groups.

### MRI acquisition

MRI was performed in the ON-state with a 3-Tesla scanner (MAGNETOM Prisma, Siemens Healthcare, Erlangen, Germany) with a 64-channel head and neck coil. T1‐weighted (T1w) images were acquired with three‐dimensional (3D) magnetization‐prepared 180° radio‐frequency pulses and a rapid gradient‐echo (MP‐RAGE) sequence (repetition time: 2500 ms; echo time: 2.82 ms; flip angle: 7°, TI = 1100 ms; GRAPPA factor = 2; 1.0 mm isotropic voxels; 192 contiguous sagittal slices). DTI/DMI sequences were acquired with the following parameters: axial orientation, 42 slices, voxel size 1.5 × 1.5 × 3 mm^3^, TR 2800 ms, TE 88 ms, bandwidth 1778 Hz, flip angle 90°, simultaneous multi-band acceleration factor 2, GRAPPA factor 2, 65 diffusion-encoding gradient directions, 15 non-diffusion weighted images, 2 × 58 images with b-factors 1000 and 2000 s/mm^2^; acquisition time was 6:22 min.

### Calculation of DMI parameters and spatial normalization

All data processing was performed using our in-house platform NORA (www.nora-imaging.org). Pre-processing of diffusion-weighted images included a de-noising step^51^, followed by the correction of Gibbs-ringing artifacts^52^ and final upsampling to an isotropic resolution of 1.5 mm^3^.

Microstructural diffusion metrics based on a three-compartment diffusion model were estimated using a Bayesian approach^12^, with the aim of determining (I) the free water/CSF fraction (V-CSF), (II) volume fraction within neuronal processes (i.e. axons and dendrites; V-intra), with an almost one-dimensional molecule diffusion due to tight membrane borders, (III) volume fraction outside the neuronal processes (V-extra), corresponding to the cellular compartment and extracellular matrix. T1w-imaging datasets were automatically segmented into white matter, gray matter and cerebrospinal fluid (CSF) using CAT12 (http://www.neuro.uni-jena.de/cat/), and DMI images were co-registered to the T1w images.

To investigate DMI parameters in the voxel-wise analyses, images were spatially normalized by CAT12 using the DARTEL (diffeomorphic anatomical registration via exponentiated lie algebra) method^53^. The quantitative DMI maps were then diffeomorphically warped to MNI space.

The validity of co-registration between DMI images, T1w and binary masks was manually confirmed. Further quality control was performed by visually inspecting each individual DMI dataset and CAT12 segmentation.

#### Voxel-wise comparison of PD and HC

To reveal the spatial distribution of microstructural alterations in PD, voxel-based group comparisons of DMI parameters were carried out using a parametric multiple regression model, with age and sex as nuisance covariates implemented in the Statistical Parametric Mapping-Voxel-Based Morphometry (SPM-VBM) 8-Toolbox. For this purpose, images were smoothed with a 3-mm full-width at half-maximum (FWHM) of the Gaussian kernel. Analyses were confined to the infratentorial parenchyma that extended to the basal ganglia as pathognomonic regions of the disease^2^. Significant group effects were adjusted for multiple comparisons using a false discovery rate (FDR) of 5%.

#### ROI-based group comparison of PD and HC groups

Because neuropathological findings in patients with PD are indicative of degeneration in the SN and putamen^2^, microstructural alterations in these two regions were assessed in PD and HC groups by extracting DMI parameters using a probabilistic atlas-based approach^17,18^.

#### ROI-based analyses of DMI and clinical parameters

A further association between microstructural alterations in the SN and putamen and clinical parameters in vivo was considered by correlating DMI parameters with MDS-UPDRS III findings in the OFF and ON-states, as well as with the levodopa-response.

### Statistical analyses

Statistical analyses were performed using R (version 4.1.0, https://www.R-project.org/). Data are presented as the mean and standard deviation for continuous variables, and as absolute frequencies and percentages for categorical variables. Shapiro-Wilk test was used to assess normal distribution of data. Group differences were assessed by applying the chi-squared test and a 2-sample t-test to the data. Intergroup differences were assessed using age and sex-adjusted analysis of covariance (ANCOVA), followed by Tukey’s honest significance test. We estimated the effect sizes for pairwise comparisons with Cohen’s d. Linear regression models controlling for age and sex were used to test the strength of the relationship between MDS-UPDRS III-scores in OFF- and ON-states as well as the levodopa response as response variables and DMI parameters in the respective atlas-derived ROIs contralateral to the more affected side as explanatory variables. Multicollinearity was assessed using the variance inflation factor (VIF) in the “Performance” package^54^. We performed a complete case analysis for all models and examined residuals for internal validation. Since all analyses were exploratory in nature, *p*-values were not corrected for multiple comparisons. The significance threshold was set to *p* < 0.05.

## Supplementary information

Supplementary Material File

## Data Availability

The dataset is available from the corresponding author upon reasonable request and approval by the local ethics committee. The approval of the local ethics committee is obtained on a case-by-case basis by the corresponding author.

## Code availability

The R code used in our analysis is available from the corresponding author upon reasonable request.
