# Supplementary material for: Disentangling nigral and putaminal contribution to motor impairment and levodopa response in Parkinson’s disease
Source: NPJ Parkinsons Dis. 2022 Oct 14;8:132. doi: 10.1038/s41531-022-00401-z (PMC9568583; doi:10.1038/s41531-022-00401-z)
Supplement: Supplementary file 1 — Supplementary Material File [file 41531_2022_401_MOESM1_ESM.pdf]

Disentangling Nigral and Putaminal Contribution to Motor Impairment and Levodopa Response in Parkinson's Disease

Nils Schröter, MD[1]; Michel Rijntjes, MD[1]; Horst Urbach, MD[2]; Cornelius Weiller, MD[1]; Martin Treppner[3]; Elias Kellner, PhD[4]; Wolfgang H. Jost, MD[5]; Bastian E. A. Sajonz, MD, MSc[6]; Marco Reisert, PhD[4,6]; Jonas Hosp, MD[1]; Alexander Rau, MD[2,7]

[1]Department of Neurology and Clinical Neuroscience, Medical Center – University of Freiburg, Faculty of Medicine, University of Freiburg, Freiburg, Germany

[2]Department of Neuroradiology, Medical Center – University of Freiburg, Faculty of Medicine, University of Freiburg, Freiburg, Germany

[3]Institute of Medical Biometry and Statistics, Medical Center - University of Freiburg, Faculty of Medicine, University of Freiburg, Freiburg, Germany

[4]Department of Medical Physics, Medical Center – University of Freiburg, Faculty of Medicine, University of Freiburg, Freiburg, Germany

[5]Parkinson-Klinik Ortenau, Wolfach, Germany

[6]Department of Stereotactic and Functional Neurosurgery, Medical Center - University of Freiburg, Faculty of Medicine, University of Freiburg, Freiburg, Germany

[7]Department of Diagnostic and Interventional Radiology, Medical Center - University of Freiburg, Faculty of Medicine, University of Freiburg, Freiburg, Germany

SUPPLEMENTARY RESULTS

Voxel-wise group comparisons

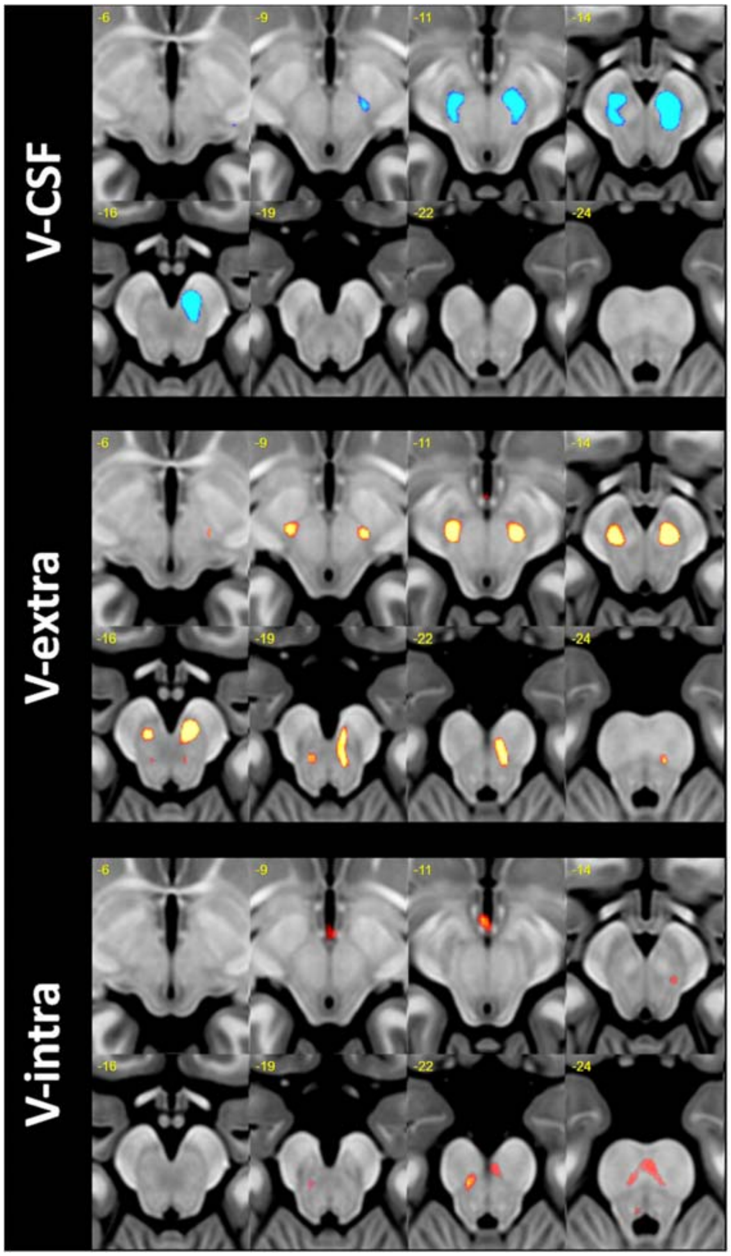

Supplementary Figure 1 Voxel-wise group comparison of DMI parameters between patients with PD (n=108) and healthy controls (n=35) superimposed on a T1w MRI template in standard space. The analysis controlled for age and sex, and a 5% false discovery rate (FDR) was applied.
Significant differences are given for the free interstitial fluid fraction (V-CSF; *upper field*), the extra-axonal compartment fraction (V-extra; *middle field*), and the intra-axonal compartment fraction (V-intra; *lower field*). The left side of the image corresponds to right side of the patient's body; numbers denote the axial (z) position in millimetres. PD, Parkinson's Disease

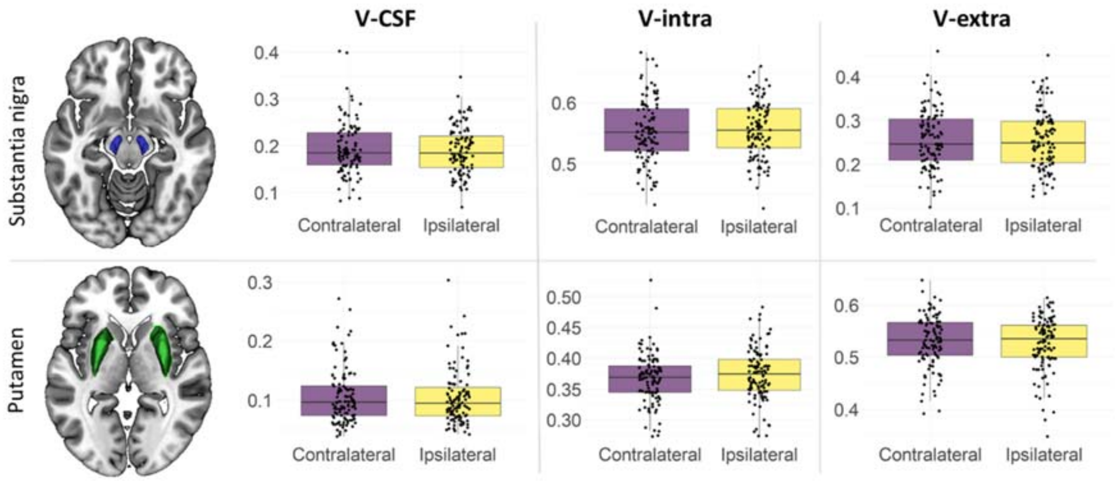

Supplementary Figure 2 DMI metrics in the substantia nigra and the putamen in Parkinson´s disease ipsilateral and contralateral to the predominant motor symptom side. No significant asymmetries were found.

Supplementary Table 1

Regression results for V-CSF in the substantia nigra

Criterion	Predictor	b	b 95% CI [LL, UL]	sr^2	sr^2 95% CI [LL, UL]	Fit
MDS-UPDRS, OFF state	(Intercept)	32.43*	[2.88, 61.98]			
	V-CSF in substantia nigra	50.65	[-16.51, 117.81]	.02	[-.04, .09]	
	Age	0.08	[-0.36, 0.53]	.00	[-.01, .02]	
	Sex: Male	0.12	[-7.59, 7.82]	.00	[-.00, .00]	
						R^2 = .031 95% CI[.00,.10]
MDS-UPDRS, ON state	(Intercept)	6.51	[-13.04, 26.05]			
	V-CSF in substantia nigra	65.79**	[21.41, 110.17]	.08	[-.02, .19]	
	Age	0.10	[-0.20, 0.40]	.00	[-.02, .03]	
	Sex: Male	-1.17	[-6.30, 3.96]	.00	[-.01, .02]	
						R^2 = .107* 95% CI[.00,.21]
Levodopa response	(Intercept)	83.61**	[56.07, 111.16]			
	V-CSF in substantia nigra	-83.66**	[-146.27, -21.05]	.07	[-.03, .16]	
	Age	-0.37	[-0.78, 0.05]	.03	[-.03, .09]	
	Sex: Male	0.54	[-6.64, 7.72]	.00	[-.01, .01]	
						R^2 = .129** 95% CI[.01,.24]

Note. A significant *b*-weight indicates that the semi-partial correlation is also significant, where *b* represents the non-standardized regression weights. sr^2 represents the semi-partial correlation squared. *LL* and *UL* indicate the lower and upper limits of a confidence interval, respectively.
* indicates p < 0.05. ** indicates p < 0.01.

Supplementary Table 2

Regression results for V-CSF in the putamen

**Criterion**	**Predictor**	***b***	***b* 95% CI [LL, UL]**	***sr*^{2}**	***sr*^{2} 95% CI [LL, UL]**	**Fit**
MDS-UPDRS, OFF state	(Intercept)	38.65*	[8.60, 68.70]			
	V-CSF in Putamen	39.55	[-52.35, 131.46]	.01	[-.03, .04]	
	Age	0.07	[-0.43, 0.56]	.00	[-.01, .01]	
	Sex: Male	0.60	[-7.13, 8.34]	.00	[-.01, .01]	
						R^2 = .015 95% CI[.00,.06]
MDS-UPDRS, ON state	(Intercept)	17.29	[-2.74, 37.32]			
	V-CSF in Putamen	86.00**	[25.71, 146.28]	.08	[-.02, .18]	
	Age	-0.01	[-0.35, 0.32]	.00	[-.00, .00]	
	Sex: Male	-0.82	[-5.94, 4.30]	.00	[-.01, .01]	
						R^2 = .101* 95% CI[.00,.21]
Levodopa response	(Intercept)	65.89**	[39.70, 92.08]			
	V-CSF in Putamen	-176.56**	[-256.67, -96.44]	.16	[.03, .30]	
	Age	-0.06	[-0.49, 0.37]	.00	[-.01, .01]	
	Sex: Male	0.83	[-5.91, 7.57]	.00	[-.01, .01]	
						R^2 = .226** 95% CI[.07,.35]

Note. A significant *b*-weight indicates that the semi-partial correlation is also significant, where *b* represents the non-standardized regression weights. *sr*^{2} represents the semi-partial correlation squared. *LL* and *UL* indicate the lower and upper limits of a confidence interval, respectively.
* indicates p < 0.05. ** indicates p < 0.01.

Supplementary Table 3

Regression results for V-CSF in the substantia nigra and putamen

Criterion	Predictor	*b*	*b* 95% CI [LL, UL]	sr^2	sr^2 95% CI [LL, UL]	Fit
MDS-UPDRS, OFF state	(Intercept)	34.33*	[3.70, 64.96]			
	V-CSF in Putamen	23.83	[-70.73, 118.39]	.00	[-.02, .02]	
	V-CSF in substantia nigra	46.25	[-23.42, 115.93]	.02	[-.04, .07]	
	Age	0.03	[-0.47, 0.52]	.00	[-.00, .00]	
	Sex: Male	-0.04	[-7.80, 7.72]	.00	[-.00, .00]	
						R^2 = .034 95% CI[.00,.09]
MDS-UPDRS, ON state	(Intercept)	12.66	[-7.33, 32.64]			
	V-CSF in Putamen	66.64*	[5.38, 127.91]	.04	[-.03, .12]	
	V-CSF in substantia nigra	52.36*	[7.11, 97.60]	.05	[-.03, .13]	
	Age	-0.06	[-0.38, 0.27]	.00	[-.01, .01]	
	Sex: Male	-1.56	[-6.60, 3.48]	.00	[-.02, .03]	
						R^2 = .151** 95% CI[.02,.26]
Levodopa response	(Intercept)	70.97**	[44.49, 97.45]			
	V-CSF in Putamen	-158.03**	[-239.79, -76.27]	.12	[.01, .24]	
	V-CSF in substantia nigra	-54.49	[-114.73, 5.75]	.03	[-.03, .08]	
	Age	-0.02	[-0.44, 0.41]	.00	[-.00, .00]	
	Sex: Male	1.59	[-5.12, 8.30]	.00	[-.01, .02]	
						R^2 = .253** 95% CI[.09,.37]

Note. A significant *b*-weight indicates that the semi-partial correlation is also significant, where *b* represents the non-standardized regression weights. sr^2 represents the semi-partial correlation squared. *LL* and *UL* indicate the lower and

upper limits of a confidence interval, respectively.
* indicates p < 0.05. ** indicates p < 0.01.

Supplementary Table 4

Regression results for V-extra in the substantia nigra

Criterion	Predictor	*b*	*b* 95% CI [LL, UL]	sr^2	sr^2 95% CI [LL, UL]	Fit
MDS-UPDRS, OFF state	(Intercept)	44.20*	[9.51, 78.89]			
	V-extra in substantia nigra	-27.03	[-87.71, 33.66]	.01	[-.03, .05]	
	Age	0.16	[-0.28, 0.60]	.01	[-.02, .04]	
	Sex: Male	-0.29	[-8.49, 7.91]	.00	[-.00, .00]	
						R^2 = .016 95% CI[.00,.07]
MDS-UPDRS, ON state	(Intercept)	26.62*	[3.44, 49.80]			
	V-extra in substantia nigra	-53.28*	[-94.08, -12.48]	.07	[-.03, .16]	
	Age	0.21	[-0.08, 0.51]	.02	[-.03, .08]	
	Sex: Male	-2.70	[-8.22, 2.82]	.01	[-.03, .05]	
						R^2 = .090* 95% CI[.00,.19]
Levodopa response	(Intercept)	60.56**	[27.94, 93.17]			
	V-extra in substantia nigra	56.55	[-0.50, 113.61]	.04	[-.04, .11]	
	Age	-0.50*	[-0.90, -0.09]	.06	[-.03, .15]	
	Sex: Male	1.77	[-5.94, 9.48]	.00	[-.02, .02]	
						R^2 = .100* 95% CI[.00,.20]

Note. A significant *b*-weight indicates that the semi-partial correlation is also significant, where *b* represents the non-standardized regression weights. sr^2 represents the semi-partial correlation squared. *LL* and *UL* indicate the lower and upper limits of a confidence interval, respectively.

* indicates $p < 0.05$. ** indicates $p < 0.01$.

Supplementary Table 5

Regression results for V-extra in the putamen

Criterion	Predictor	*b*	*b* 95% CI [LL, UL]	sr^2	sr^2 95% CI [LL, UL]	Fit
MDS-UPDRS, OFF state	(Intercept)	63.49	[-2.27, 129.26]			
	V-extra in Putamen	-41.43	[-130.08, 47.22]	.01	[-.03, .05]	
	Age	0.09	[-0.37, 0.55]	.00	[-.01, .02]	
	Sex: Male	0.33	[-7.47, 8.13]	.00	[-.00, .00]	
						R^2 = .016 95% CI[.00,.07]
MDS-UPDRS, ON state	(Intercept)	57.40*	[13.24, 101.57]			
	V-extra in Putamen	-69.93*	[-128.73, -11.14]	.06	[-.03, .14]	
	Age	0.09	[-0.23, 0.40]	.00	[-.02, .02]	
	Sex: Male	-1.21	[-6.46, 4.04]	.00	[-.02, .02]	
						R^2 = .079 95% CI[.00,.18]
Levodopa response	(Intercept)	-3.40	[-63.66, 56.85]			
	V-extra in Putamen	122.23**	[41.00, 203.46]	.08	[-.02, .19]	
	Age	-0.29	[-0.71, 0.14]	.02	[-.03, .07]	
	Sex: Male	1.04	[-6.10, 8.19]	.00	[-.01, .01]	
						R^2 = .146** 95% CI[.02,.26]

Note. A significant *b*-weight indicates that the semi-partial correlation is also significant, where *b* represents the non-standardized regression weights. sr^2 represents the semi-partial correlation squared. *LL* and *UL* indicate the lower and upper limits of a confidence interval, respectively.
* indicates p < 0.05. ** indicates p <0.01.

Supplementary Table 6

Regression results for V-extra in the substantia nigra and putamen

Criterion	Predictor	*b*	*b* 95% CI [LL, UL]	sr^2	sr^2 95% CI [LL, UL]	Fit
MDS-UPDRS, OFF state	(Intercept)	64.02	[-1.98, 130.03]			
	V-extra in Putamen	-32.81	[-125.65, 60.02]	.01	[-.02, .03]	
	V-extra in substantia nigra	-20.60	[-84.12, 42.93]	.00	[-.02, .03]	
	Age	0.10	[-0.36, 0.57]	.00	[-.02, .02]	
	Sex: Male	-0.51	[-8.76, 7.74]	.00	[-.00, .01]	
						R^2 = .021 95% CI[.00,.06]
MDS-UPDRS, ON state	(Intercept)	57.84**	[14.36, 101.33]			
	V-extra in Putamen	-51.40	[-112.20, 9.40]	.03	[-.03, .09]	
	V-extra in substantia nigra	-42.31	[-84.75, 0.12]	.04	[-.03, .11]	
	Age	0.12	[-0.19, 0.43]	.01	[-.02, .03]	
	Sex: Male	-3.03	[-8.50, 2.45]	.01	[-.03, .05]	
						R^2 = .117* 95% CI[.00,.22]
Levodopa response	(Intercept)	-4.31	[-64.44, 55.81]			
	V-extra in Putamen	107.37*	[22.80, 191.93]	.06	[-.03, .15]	
	V-extra in substantia nigra	35.52	[-22.34, 93.38]	.01	[-.03, .06]	
	Age	-0.31	[-0.73, 0.12]	.02	[-.03, .07]	
	Sex: Male	2.49	[-5.02, 10.01]	.00	[-.02, .03]	
						R^2 = .160** 95% CI[.02,.27]

Note. A significant *b*-weight indicates that the semi-partial correlation is also significant, where *b* represents the non-standardized regression weights. sr^2 represents the semi-partial correlation squared. *LL* and *UL* indicate the lower and

upper limits of a confidence interval, respectively.
* indicates $p < 0.05$. ** indicates $p < 0.01$.

Supplementary Table 7

Regression results for V-intra in the substantia nigra

Criterion	Predictor	*b*	*b* 95% CI [LL, UL]	sr^2	sr^2 95% CI [LL, UL]	Fit
MDS-UPDRS, OFF state	(Intercept)	50.41	[-4.90, 105.72]			
	V-intra in substantia nigra	-23.60	[-100.26, 53.06]	.00	[-.02, .03]	
	Age	0.14	[-0.31, 0.58]	.00	[-.02, .03]	
	Sex: Male	1.82	[-6.32, 9.96]	.00	[-.02, .02]	
						R^2 = .011 95% CI[.00,.05]
MDS-UPDRS, ON state	(Intercept)	16.76	[-22.72, 56.25]			
	V-intra in substantia nigra	-10.31	[-64.97, 44.35]	.00	[-.01, .02]	
	Age	0.21	[-0.10, 0.52]	.02	[-.03, .07]	
	Sex: Male	0.37	[-5.31, 6.05]	.00	[-.01, .01]	
						R^2 = .025 95% CI[.00,.09]
Levodopa response	(Intercept)	67.09*	[14.15, 120.02]			
	V-intra in substantia nigra	17.40	[-55.96, 90.76]	.00	[-.02, .02]	
	Age	-0.48*	[-0.91, -0.06]	.05	[-.03, .14]	
	Sex: Male	-1.52	[-9.32, 6.27]	.00	[-.01, .02]	
						R^2 = .063 95% CI[.00,.15]

Note. A significant *b*-weight indicates that the semi-partial correlation is also significant, where *b* represents the non-standardized regression weights. sr^2 represents the semi-partial correlation squared. *LL* and *UL* indicate the lower and upper limits of a confidence interval, respectively.
* indicates $p < 0.05$.

Supplementary Table 8

Regression results for V-intra in the putamen

Criterion	Predictor	*b*	*b* 95% CI [LL, UL]	sr^2	sr^2 95% CI [LL, UL]	Fit
MDS-UPDRS, OFF state	(Intercept)	38.94	[-13.85, 91.74]			
	V-intra in Putamen	-7.06	[-112.16, 98.03]	.00	[-.01, .01]	
	Age	0.16	[-0.29, 0.61]	.01	[-.02, .04]	
	Sex: Male	1.03	[-6.69, 8.75]	.00	[-.01, .01]	
						R^2 = .007 95% CI[.00,.04]
MDS-UPDRS, ON state	(Intercept)	20.38	[-16.65, 57.41]			
	V-intra in Putamen	-23.73	[-97.24, 49.79]	.00	[-.02, .03]	
	Age	0.20	[-0.11, 0.51]	.02	[-.03, .07]	
	Sex: Male	0.14	[-5.17, 5.45]	.00	[-.00, .00]	
						R^2 = .027 95% CI[.00,.09]
Levodopa response	(Intercept)	49.05	[-0.91, 99.02]			
	V-intra in Putamen	68.82	[-30.64, 168.28]	.02	[-.03, .07]	
	Age	-0.45*	[-0.87, -0.03]	.05	[-.04, .13]	
	Sex: Male	-1.22	[-8.53, 6.08]	.00	[-.01, .01]	
						R^2 = .080 95% CI[.00,.18]

Note. A significant *b*-weight indicates that the semi-partial correlation is also significant, where *b* represents the non-standardized regression weights. sr^2 represents the semi-partial correlation squared. *LL* and *UL* indicate the lower and upper limits of a confidence interval, respectively.
* indicates $p < 0.05$.

Supplementary Table 9

Regression results for V-intra in substantia nigra and Putamen

Criterion	Predictor	*b*	*b* 95% CI [LL, UL]	sr^2	sr^2 95% CI [LL, UL]	Fit
MDS-UPDRS, OFF state	(Intercept)	49.14	[-13.71, 112.00]			
	V-intra in Putamen	4.87	[-107.78, 117.52]	.00	[-.00, .00]	
	V-intra in substantia nigra	-24.85	[-107.19, 57.49]	.00	[-.02, .03]	
	Age	0.14	[-0.31, 0.59]	.00	[-.02, .03]	
	Sex: Male	1.84	[-6.37, 10.05]	.00	[-.02, .02]	
						R^2 = .011 95% CI[.00,.03]
MDS-UPDRS, ON state	(Intercept)	22.74	[-22.42, 67.91]			
	V-intra in Putamen	-21.49	[-99.29, 56.32]	.00	[-.02, .03]	
	V-intra in substantia nigra	-5.33	[-63.09, 52.44]	.00	[-.01, .01]	
	Age	0.20	[-0.12, 0.51]	.02	[-.03, .07]	
	Sex: Male	0.32	[-5.38, 6.03]	.00	[-.00, .00]	
						R^2 = .028 95% CI[.00,.08]
Levodopa response	(Intercept)	49.17	[-10.44, 108.78]			
	V-intra in Putamen	68.96	[-37.87, 175.78]	.02	[-.03, .07]	
	V-intra in substantia nigra	-0.29	[-78.37, 77.79]	.00	[-.00, .00]	
	Age	-0.45*	[-0.88, -0.02]	.04	[-.04, .12]	
	Sex: Male	-1.21	[-9.00, 6.57]	.00	[-.01, .01]	
						R^2 = .080 95% CI[.00,.17]

Note. A significant *b*-weight indicates that the semi-partial correlation is also

significant, where *b* represents the non-standardized regression weights. sr^2 represents the semi-partial correlation squared. *LL* and *UL* indicate the lower and upper limits of a confidence interval, respectively.
* indicates $p < 0.05$.
